# Genotype-Phenotype Correlation: A Triple DNA Mutational Event in a Boy Entering Sport Conveys an Additional Pathogenicity Risk

**DOI:** 10.3390/genes11050524

**Published:** 2020-05-08

**Authors:** Giuseppe Limongelli, Marcella Nunziato, Cristina Mazzaccara, Mariano Intrieri, Valeria D’Argenio, Maria Valeria Esposito, Emanuele Monda, Federica Di Maggio, Giulia Frisso, Francesco Salvatore

**Affiliations:** 1 Department of Translational Medical Sciences, University of Campania “Luigi Vanvitelli”, AO Colli-Monaldi Hospital, Via Leonardo Bianchi, 80131 Naples, Italy; emanuelemonda@me.com; 2 Department of Molecular Medicine and Medical Biotechnologies, University of Naples Federico II, Via Sergio Pansini, 5, 80131 Naples, Italy; nunziato@ceinge.unina.it (M.N.); cristina.mazzaccara@unina.it (C.M.); espositomari@ceinge.unina.it (M.V.E.); dimaggio@ceinge.unina.it (F.D.M.); gfrisso@unina.it (G.F.); 3 CEINGE-Advanced Biotechnologies, Via G. Salvatore 486, 80145 Naples, Italy; dargenio@ceinge.unina.it; 4 Department of Medicine and Health Sciences “Vincenzo Tiberio” and University of Molise, Campobasso, Via de Sanctis, 86100 Campobasso, Italy; intrieri@unimol.it; 5 San Raffaele Open University, Via di Val Cannuta, 247, 00166 Rome, Italy

**Keywords:** sports activities, sudden cardiac death, next-generation sequencing, genotype-phenotype correlation, gene mutations in athletes

## Abstract

The purpose of this paper is to present a clinical and laboratory study of a family, in which a 12-year-old boy was examined to assess his health status before starting competitive sports. A variety of clinical and instrumental tests were used to evaluate the status of the heart and its functions. Using Sanger sequencing (SS), we sequenced six related genes to verify suspected arrhythmogenic right ventricular cardiomyopathy (ARVC) hypothesized at the cardiac assessment and, subsequently, by a next-generation sequencing (NGS)-based multi-gene panel for more paramount genetic risk of sudden cardiac death (SCD) assessment. SS revealed two variants in the *PKP2* gene, one was inherited from the father and the other from the mother. The analysis on a large panel of genes (*n* = 138), putatively associated with sudden cardiac death, revealed, in the proband, a third variant in a different gene *(DES)* that encodes the protein desmin. Our results indicate that: i) NGS revealed a mutational event in a gene not conventionally screened as a first-line test in the presence of clinical suspicion of the arrhythmic disease; ii) a plurality of variants in different genes in the same subject (the proband) may increase the risk of heart disease; iii) in silico analysis with various methodological software and bioinformatic prediction tools indicates that the cumulative effects of the three variants in the same subject constitute an additional risk factor. This case report indicates that more pathogenic variants or likely pathogenic variants can contribute to the clinical phenotype of an individual, thereby contributing to the diagnosis and prognosis of inherited heart diseases.

## 1. Introduction

Herein, we describe the case of a young boy who was suspected of having an arrhythmogenic cardiomyopathy (AC) after a comprehensive cardiological evaluation. The cardiac abnormalities were identified during the pre-sports participation screening. The aim of this screening is to decrease, or even eliminate, sport-related injuries and death by identifying abnormal signs or symptoms of cardiovascular disease (i.e., cardiomyopathies and channelopathies) that may predispose to life-threatening ventricular arrhythmias [1,2,3]. Intense sport practice, mainly motor activity, but also creative and intellectual activities, may facilitate adverse electrical and morphological cardiac remodeling in young subjects with genetic predisposition for inherited heart disease [4]. Lastly, an Expert Consensus Statement on the Diagnosis and Management of Patients with Inherited Primary Arrhythmia Syndromes recommends forbidding strenuous or competitive sports activity to avoid or limit these tragic events [3], particularly, but not only, in symptomatic patients [5,6].

## 2. Results

### 2.1. Case Study Presentation and Clinical Description

A 12-year-old boy, who practiced amateur sports activities, underwent a medical examination to assess his health status before starting competitive sports. During the pre-participation program, the boy underwent a physical examination, spirometry, urine analysis, standard 12-lead electrocardiogram (ECG), and a stress test. The ECG showed T-wave inversion in the precordial right lead (V1, V2, and V3), which was interpreted as repolarization juvenile pattern. However, during the recovery phase of the stress test, he experienced frequent ventricular ectopics with left bundle branch block morphology and inferior axis. Therefore, to obtain a definitive diagnosis, also considering that he may become more physically active, the boy was admitted at the Inherited and Rare Cardiovascular Diseases Clinic of the University of Campania, Luigi Vanvitelli, and underwent a comprehensive cardiological evaluation, including personal and family history, physical examination, blood tests, standard 12-lead ECG, echocardiography, stress test, 24-h ECG Holter, and high resolution signal-averaged electrocardiogram (SAECG). A 24-h ECG Holter revealed many episodes of wide QRS tachycardia with spontaneous resolution and electrical alternation of the QRS.

**Figure 1:**
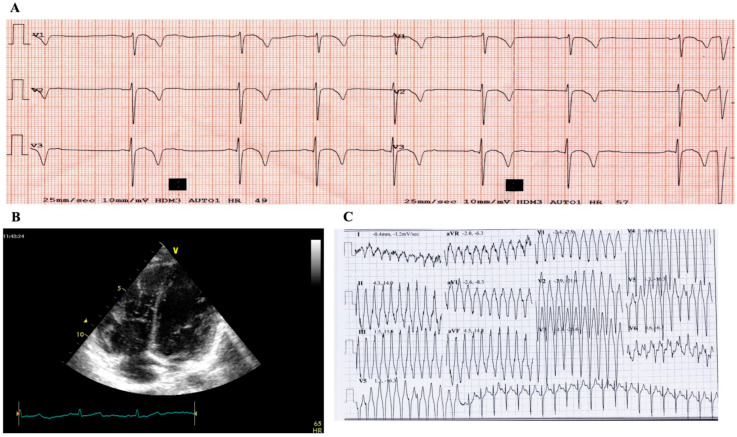
Clinical instrumental cardiac signs of the index case. (**A**) T-wave inversion (TWI) in anterior leads at electrocardiogram (ECG); (**B**) increased trabeculation of the right ventricle at echo-ultrasonography; and (**C**) non-sustained ventricular tachycardia (NSVT), with left bundle branch block morphology and inferior axis, during stress test at ECG.

The child’s mother denied a family history of cardiomyopathies or other cardiovascular diseases associated with sudden cardiac death (SCD). However, it then emerged that two maternal cousins had died suddenly. The results of the proband’s clinical biochemistry tests were normal. ECG revealed sinus rhythm at 75 bpm, with a PR interval of 120 ms and a QTc of 420 msec in the V5 lead (Figure 1A). It did not show any abnormality in morphology and/or function, except for clearly evident trabeculation at the level of mid and apical segments of the right ventricle (Figure 1B); no regional right ventricular akinesia, dyskinesia, or aneurysm was identified. During the stress test, non-sustained ventricular tachycardia (NSVT) with left bundle branch block morphology and inferior axis was observed at ECG (Figure 1C). Holter monitoring showed 1397 premature ventricular contractions (PVCs) per 24 h and 174 paired ventricular extra systoles per 24 h. The SAECG achieved normal values. 

All first-degree relatives (the parents and the brother) of the boy underwent a complete assessment: clinical evaluation, ECG, 24 h Holter monitoring, echocardiography, and SAECG. The relatives were asymptomatic on that occasion and had no ECG or echocardiographic abnormalities. The proband and relatives refused to perform cardiac magnetic resonance imaging (cMRI) for claustrophobia.

Although a putative diagnosis of AC for the proband was suspected, in order to better understand the possible molecular alterations present in this family’s members, we sequenced the DNA of the proband and those of his relatives.

### 2.2. Case Study: Clinical Molecular Biology Investigation

**Figure 2:**
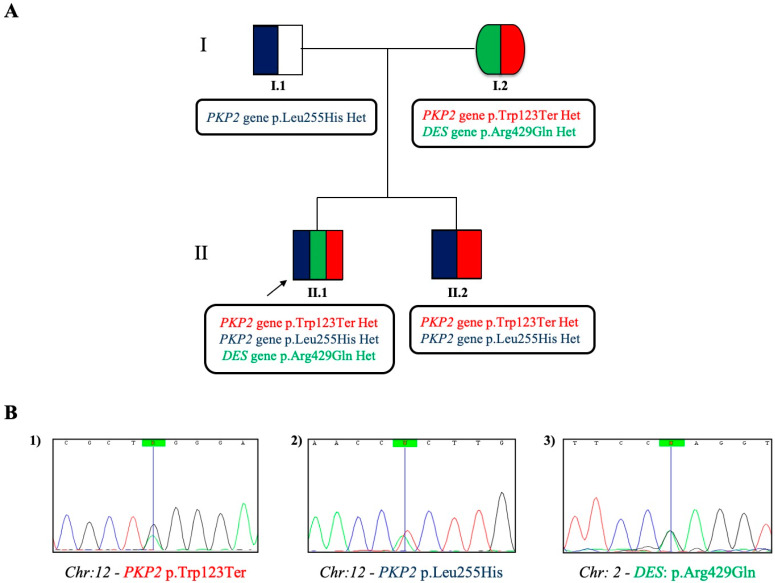
Pedigree of the analyzed family. **A**) The pedigree shows the segregation of the variants (in color) detected in the affected boy and his family. In the patient I.1, the variant carried in the heterozygous status is the novel c.764T>A (p.Leu255His) in the Plakophilin (*PKP2*) gene; I.2 carried two different variants: one in the *PKP2* gene (the c.368G>A (p.Trp123Ter)) and the other (found with next-generation sequencing (NGS) technology) in the *DES* gene (the c.1286G>A (p.Arg429Gln)), both in heterozygosity. The proband of the study (II.1) shows all the three variants cited above in the parents; his younger brother (II.2) shows only the two variants in the *PKP2* gene. **B**) Electropherograms from Sanger Sequencing for 1) *PKP2*: c.368G>A, 2) *PKP2*: c.764T>A, and 3) *DES*: c.1286G>A variants, respectively.

Informed written consent for DNA analysis was obtained from the proband, his parents, and his younger brother, according to the procedure established by the Second Helsinki Declaration and according to Italian and local Regulations (Ethic Committee of the Monaldi Hospital-Naples-#189 15/03/2009). Genomic DNA was isolated from peripheral whole blood, as described elsewhere [7]. The patient’s DNA was amplified by polymerase chain reaction (PCR) for all exons, the flanking regions ranged from a minimum of 25 bp, and a maximum of 220 bp of the six following most often involved genes in arrhythmogenic right ventricular cardiomyopathy (ARVC) disease: Plakophilin (*PKP2*), Desmoplakin (*DSP*), Desmoglein-2 (*DSG2*), Desmocollin-2 (*DSC2*), Ryanodine Receptor 2 (*RYR2*), Junction Plakoglobin (*JUP*), using previously reported protocols [8]. Automated Sanger DNA sequencing with the ABI PRISM 3100 DNA Analyzer (Applied Biosystems Big-Dye Terminator v1.1 cycle sequencing kit, Life Technologies, Grand Island, NY, USA) was used after PCR reactions. The relatives of the proband were screened for the variants found in the proband by SS. Analysis of the six genes showed that the proband was a composite heterozygote for two variants in the *PKP2* gene: c.368G>A (p.Trp123Ter) and c.764T>A (p.Leu255His) (Figure 2). The first variant (p.Trp123Ter) was an AC-associated variant (pathogenic, as reported in the ClinVar database), previously described in a large AC family, in which the authors studied the segregation of the variant in a three generation family pedigree and found three subjects affected by AC who carried the variant. One of the three died at the age of 18 years old, while the other two carriers of the variant were, at that moment, not clinically affected by AC (one was 5 years old at the diagnosis) [9]. The latter (p.Leu255His) is a novel variant, classified as variant of unknown significance (VUS) by the American College of Medical Genetics and Genomics (ACMG) [10]. We performed a complete in silico prediction using the VarSome tool: The variant is not present in gnomAD exomes and gnomAD genomes (this constitutes an ACMG Pathogenic Moderate rule: The Genome Aggregation Database (gnomAD) is a resource which aggregates both exome and genome sequencing data from numerous projects. The database contains 125,748 exome sequences and 15,708 whole-genome sequences from unrelated individuals sequenced). Moreover, the novel variant was indicated as pathogenic by 10 predictions tools (considering also the uncertain prediction of the DANN score, which is equal to 0.9848 and constitutes, for ACMG, a Pathogenic Supporting rule —the value range is 0 to 1, with 1 given to the variants predicted to be the most damaging [11]—and FATHMM-MKL, DEOGEN2, EIGEN, M-CAP, MutationAssessor, MutationTaster, SIFT, MVP, REVEL), vs only one benign prediction from the PrimateAI tool. Based on this, and because the two variants in the *PKP2* gene were on different alleles (one variant was inherited from the mother and the other from the father), considering the presence of a certainly pathogenic variant (the stop codon) and another one variant not yet defined but with a hint of pathogenicity, it is possible, in our opinion, that the protein produced (plakophilin) may not work properly. Thus, it is likely possible that the double mutation in the same gene may confer additional pathogenicity. Segregation analysis in the parents showed that the two variants were in trans: the parents were both carriers of one *PKP2* variant; however, they both did not show any clinical signs or symptoms of AC, though the diseases were prevalently considered as dominant. The absence of clinical phenotype (negative ECG and imaging) in the mother, carrying the stop codon variant in *PKP2* gene, could be explained by the presence of protective factors not known at the time or by the lack of protective genetic assessment existing in her son genome. However, it was suggested that she be followed-up to exclude any age-related phenotype. 

The genetic analysis was also conducted in the proband’s younger brother, practicing amateur sports (see Figure 2). However, subsequently, this younger boy was forced to quit practicing soccer at a competitive level by both clinical and genetic evidences and through multi-disciplinary medical counseling.

To better understand the genotype–phenotype correlation of this intriguing family case-study (intriguing, particularly in view of the pathogenic mutational event in the proband’s mother), we performed genetic analysis by next generation sequencing (NGS) to verify the presence of a pro-arrhythmogenic genetic background by analyzing a greater number of genes [12]. To this aim, we used a home-made multi-gene panel, constituted of 138 target genes, including 3009 target regions by the use of 54,656 probes for a total size of 2147 million base pairs. Our customized panel included all coding exons for each gene and 50 bp at exon boundaries on each side (5’ and 3’). It included 49 genes related principally to structural cardiomyopathies; 40 genes related to electric alterations; 29 genes related to both of the above categories; and 20 genes related to other hereditary heart diseases (e.g., metabolic syndromes), not included in the other above-mentioned three groups of genes and principally related to sudden cardiac death risk. Libraries were prepared as previously described [13]. The results of the presence of genetic alterations, after the analysis with this gene’s panel for SCD risk, confirmed the Sanger methodology for the *PKP2* gene variants and revealed, in addition, the presence of a rare variant in the *DES* gene (c.1286G>A p.Arg429Gln), which was maternally inherited (see Figure 2) and absent in his younger brother. This variant was classified as “likely pathogenic” based on ACMG guidelines and many other prediction tools. More specifically, the variant in DES gene was classified as “likely pathogenic” because it fulfilled four different ACMG criteria: (i) Pathogenic moderate rule due to the region where it is located: “Tail”, which presents 12 pathogenic and only 5 benign variants (data from Uniprot, https://www.uniprot.org searching DESM_HUMAN); (ii) for the really low allele frequency (0.0000517 less than 0.0001 threshold for recessive gene *DES*) in GnomAD exomes; (iii) pathogenic supporting rule because 76 out of 78 non-VUS missense variants in the gene are known to be pathogenic (ClinVar database); and (iv) for in silico prediction tools: there are eight pathogenic predictions from DANN, EIGEN, FATHMM-MKL, M-CAP, MutationAssessor, MutationTaster, PrimateAI, and SIFT vs only three benign predictions from DEOGEN2, MVP, and REVEL. Given this, we believe that the cumulative effect of the three variants found may contribute to a more severe AC phenotype.

## 3. Discussion

The negative influence of strenuous physical activity in individuals carrying “silent mutations” for hereditary cardiomyopathies and channelopathies is a topic of great interest in cardiology and sport medicine literature [14,15,16]. Indeed, physical exercise, especially strenuous physical exercise, is associated with an earlier onset of the phenotype of these diseases, which is often silent; it is also associated with worse phenotypes, and, most importantly, with an increased risk of SCD due to arrhythmic risk and/or progression of cardiac dysfunction [14,15,16,17]. Therefore, a correct and early diagnosis of these diseases is very important, especially in young individuals approaching competitive sports [18,19]. The clinical diagnosis of inherited heart diseases is often challenging because of the non-specific nature of these conditions and the broad spectrum of phenotypes. Moreover, very often, the absence of signs and symptoms at cardiological examination, even when predisposing mutations are present, can confound the clinical assessment and management of these patients and their families [20,21]. Consequently, the best diagnostic approach is to combine diverse diagnostic tests, particularly in patients affected by AC. Nearly a decade ago, Marcus et al. [22] proposed a revision of International Task Force Criteria for the clinical diagnosis of arrhythmogenic right ventricular cardiomyopathy/dysplasia to include emerging diagnostic modalities and advances in the genetics of this disease. In our opinion, genetic testing is rather important, complementary, and also a priori or contemporary to other diagnostic approaches, as is highlighted in this case report and recently suggested [23,24], at least in the proband of the clinical-instrumental approaches. There is an open debate regarding which cardiac test should be performed in athletes before they start practicing sports activities to avoid greater risk to the young athletes, particularly given the less stringent regulations in USA and Canada, compared to Europe [25,26,27]. However, more recent regulatory indications seem to be more cautionary, by looking more carefully to functional and genetic testing [25,26,27,28,29]. The case reported herein supports this more cautious approach, since it indicates that the triple mutational event in the proband, after a more thorough DNA sequencing approach, revealed a more severe and early presence of DNA alteration and, consequently, a greater risk of developing a life-threatening heart muscle disorder. The single mutated gene did not show (also in presence of a likely pathogenic or pathogenic variant) a high-risk phenotypic situation in the carriers (see father and mother). This intriguing finding supports the idea that, for these diseases, there is not always a simple dominant-type situation to face, but the disease to be evident appears to need two mutations that is a recessive situation. A third mutation in another gene, such as the *DES* gene, renders the phenotype more eager to show overt symptoms or signs of electrical and morphologic adverse cardiac remodeling. Our case report indicates that genetic testing plays an important role in the diagnosis of inherited cardiovascular disease. Indeed, for the diagnosis of AC, the Revised Task Force criteria classifies as a major criterion in the presence of at least a heterozygous pathogenic variant in one of the genes known to be associated with this pathological condition [22].

Arrhythmogenic cardiomyopathy is a group of hereditary cardiomyopathies, histologically characterized by progressive fibrofatty replacement of the myocardium. Generally, AC manifests as ventricular arrhythmias: The most common arrhythmia is sustained or non-sustained monomorphic VT, which is very often related to physical exercise. Moreover, SCD due to an arrhythmic event may be the first presentation of the disease. Heart failure can occur at advanced stages of the disease. The prevalence of AC is estimated to range between 1:5000 and 1:1000 in the general population, but it is greater in some geographic areas. Indeed, it can be as high as 0.4–0.8% in Italy and on the Island of Naxos (Greece) [30,31]. AC is caused by mutations in desmosome genes in about 40–50% of cases [32,33]. The advent of molecular genetics had a tremendous impact on disease diagnosis. The estimate of digenic/biallelic inheritance varies from 4–47% [34,35,36,37,38]. In some studies, digenic/biallelic carriers have a more severe arrhythmia phenotype [39]. Multiple mutations in this case family study are found in the proband with respect to relatives, which suspect and extend the possible association of more mutations in any subject that may produce a significant increase in the risk of disease expression (as also described previously).

## 4. Conclusions and Perspectives

As genetic analysis becomes faster and cheaper, clinicians will increasingly benefit from predictive medicine, also for heart disease evaluation, therapeutic decision-making, or preventive measures of monitoring and surveillance of predisposed individuals. Genotype–phenotype observation will always appear more strongly associated as we enhance understanding of the patient’s disease. However, we still need to better understand how much each single variant contributes to the overall expression of the disease. Although the pattern of at-risk gene alterations at a multi-gene level marked a turning point in disease diagnosis, the whole genome amplification pattern, when available, together with clinical and instrumental approaches, will establish the more precise individualized medicine devoted to each subject for a preventive and closer monitoring approach. We believe that the interpretation of variants should be verified approximately every 6 months (all the variants in the present study were checked at study onset and last checked in January 2020). Indeed, it is not uncommon that the interpretation of variants changes after further studies. Moreover, we are fully aware that in silico predictions are not enough to be certain of the variants’ pathogenicity and in vitro functional studies would be needed to have more conclusive information on each identified variant [40].
